# Integrative analyses reveal a long noncoding RNA-mediated sponge regulatory network in prostate cancer

**DOI:** 10.1038/ncomms10982

**Published:** 2016-03-15

**Authors:** Zhou Du, Tong Sun, Ezgi Hacisuleyman, Teng Fei, Xiaodong Wang, Myles Brown, John L. Rinn, Mary Gwo-Shu Lee, Yiwen Chen, Philip W. Kantoff, X. Shirley Liu

**Affiliations:** 1 Shanghai Key Laboratory of Tuberculosis, Shanghai Pulmonary Hospital, Shanghai 200433, China; 2 Department of Bioinformatics, School of Life Sciences and Technology, Tongji University, Shanghai 200092, China; 3 Department of Medical Oncology, Dana-Farber Cancer Institute, Boston, Massachusetts 02215, USA; 4 Harvard Medical School, Boston, Massachusetts 02215, USA; 5 Department of Pathology, University of Massachusetts Medical School, Worcester, Massachusetts 01655, USA; 6 Department of Molecular and Cellular Biology, Harvard University, Cambridge, Massachusetts 02138, USA; 7 Department of Stem Cell and Regenerative Biology, Harvard University, Cambridge, Massachusetts 02138, USA; 8 Broad Institute of Massachusetts Institute of Technology and Harvard, Cambridge, Massachusetts 02142, USA; 9 Center for Functional Cancer Epigenetics, Dana-Farber Cancer Institute, Boston, Massachusetts 02215, USA; 10 Department of Pathology, Beth Israel Deaconess Medical Center, Boston, Massachusetts, USA; 11 Department of Bioinformatics and Computational Biology, Division of Quantitative Sciences, University of Texas MD Anderson Cancer Center, Houston, Texas 77030, USA; 12 Department of Medicine Memorial Sloan Kettering Cancer Center 1275 York Avenue, New York, New York 10065, USA; 13 Department of Biostatistics and Computational Biology, Dana-Farber Cancer Institute and Harvard School of Public Health, Boston, Massachusetts 02215, USA

## Abstract

Mounting evidence suggests that long noncoding RNAs (lncRNAs) can function as microRNA sponges and compete for microRNA binding to protein-coding transcripts. However, the prevalence, functional significance and targets of lncRNA-mediated sponge regulation of cancer are mostly unknown. Here we identify a lncRNA-mediated sponge regulatory network that affects the expression of many protein-coding prostate cancer driver genes, by integrating analysis of sequence features and gene expression profiles of both lncRNAs and protein-coding genes in tumours. We confirm the tumour-suppressive function of two lncRNAs (TUG1 and CTB-89H12.4) and their regulation of PTEN expression in prostate cancer. Surprisingly, one of the two lncRNAs, TUG1, was previously known for its function in polycomb repressive complex 2 (PRC2)-mediated transcriptional regulation, suggesting its sub-cellular localization-dependent function. Our findings not only suggest an important role of lncRNA-mediated sponge regulation in cancer, but also underscore the critical influence of cytoplasmic localization on the efficacy of a sponge lncRNA.

## Abstract (web summary)

Long non-coding RNAs (lncRNA; >200 base pair nucleic acids with little protein-coding capacity) are emerging as potentially important regulators of oncogenesis. Here the authors show tumour suppressive lncRNA sponge function for the protein products of prostate cancer driver genes.

## Graphical abstract

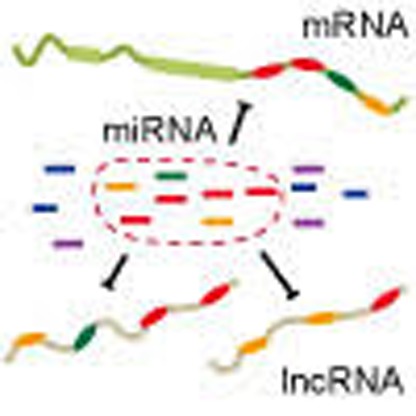

Approximately 70% of the human genome is transcribed, but less than 2% of the genome encodes protein. On the basis of size, noncoding RNAs (ncRNAs) can be classified as small (≤200 base pairs) or long ncRNAs (lncRNA; >200 base pairs). The human genome encodes around ten thousand lncRNA genes^1^,^2^,^3^ and, similar to protein-coding genes (PCGs), some lncRNAs can mediate oncogenesis or tumour suppression and are, therefore, a potential new class of cancer therapeutic targets^4^. Despite this relevance to cancer, only a handful of lncRNAs have been functionally characterized.

An important class of small ncRNAs are ∼22 nucleotide (in mammals) microRNAs (miRNAs) that are derived from hairpin precursors^5^. These RNAs guide the RNA-induced silencing complex (RISC) to miRNA response elements (MREs) on target transcripts to post-transcriptionally regulate gene expression via transcript degradation or translation inhibition^5^. Each miRNA can target multiple target transcripts and those RNAs that share the same MREs (that is, targeted by the same miRNA or the same miRNA family) are reported to influence the expression of each other by competing for miRNA binding^6^,^7^. RNAs involved in this type of miRNA-dependent regulation have been referred to as miRNA sponges^6^,^7^, target mimics^8^ or competing endogenous RNAs (if they are endogenous to the genome)^9^.

In one study, a synthetic miRNA sponge carrying engineered MREs was ectopically expressed to competitively inhibit endogenous miRNA activity^7^. The first reported naturally occurring noncoding miRNA sponge, IPS1 from *Arabidopsis thaliana*, sequesters the phosphate (Pi) starvation-induced miRNA miR-399 and modulates the shoot Pi content^8^. Since this discovery, other naturally occurring noncoding miRNA sponges have been identified as important for biological processes including muscle differentiation^10^, host–pathogen interaction^11^ and cancer^12^.

PTENP1, a pseudogene of the tumour-suppressor PTEN (phosphatase and tensin homologue), was among the first reported noncoding miRNA sponges with a function in cancer^12^. Compared with PTEN, PTENP1 has a truncated (by ∼1 kb) but highly similar 3′ region, which contains conserved target sites for the PTEN-targeting miR-17, miR-21, miR-214, miR-19 and miR-26 families. Consistent with these sequence features, PTENP1 expression is regulated by PTEN-targeting miRNAs. As a miRNA sponge, PTENP1 positively regulates PTEN expression, and the knockdown of endogenous PTENP1 promotes cancer cell proliferation, indicating the tumour-suppressive function of PTENP1 (ref. 12). Similarly, the pseudogenes of oncogenic PCGs, such as kirsten rat sarcoma viral oncogene homolog (KRAS), are also miRNA sponges^12^.

Despite identification of these pseudogenes and lncRNAs, the prevalence, functional significance of lncRNA-mediated sponge regulation and their relevant targets in human cancer are unclear. To address these questions, we systematically identify a lncRNA-mediated sponge regulatory network of protein-coding driver genes in prostate cancer by integrating sequence features and gene expression of lncRNAs and PCGs in tumours. We also validate the tumour-suppressive function of two lncRNAs predicted to serve as miRNA sponges and positively regulate PTEN expression. Our study suggests an important role of lncRNA-mediated sponge regulation in cancer and implied a therapeutical strategy of manipulating cancer gene function through modulating lncRNA-mediated sponge regulation.

## Results

### Prediction of sponge lncRNAs regulating cancer-driver genes

**Figure 1:**
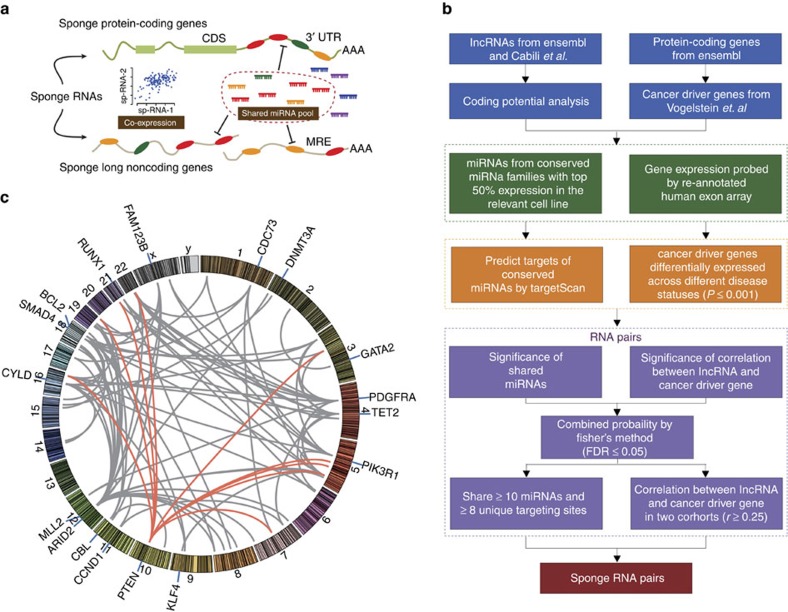
Computational prediction of sp-lncRNA regulation in prostate cancer. (**a**) The mechanism by which RNAs that are targeted by the same miRNA cross-regulate the expression of each other and the main features of the computational strategy for predicting sp-lncRNA. (**b**) The computational strategy of predicting lncRNA-mediated sponge regulation of protein-coding driver genes in prostate cancer. (**c**) A citros plot showing the computationally predicted sp-lncRNA network. The nodes represent individual genes and the edges represent the predicted regulation between sp-lncRNA and the corresponding protein-coding driver gene. FDR, false discovery rate.

Sponge-lncRNAs (sp-lncRNAs) are distinct from other regulators such as transcription factors in that they share similar miRNA regulatory programmes with their targets. Therefore, they are positive regulators of the expression of their targets (Fig. 1a), and the strength of their regulation depends on the stoichiometry of the involved miRNAs and mRNAs (Fig. 1a). We devised an integrated computational approach to predict lncRNAs that serve as sp-lncRNA for a given PCG by taking into account these characteristics (Fig. 1b, Methods). We developed a computational pipeline that repurposed the Affymetrix exon array probes for interrogating lncRNA expression^13^. Although lncRNAs were not the originally intended targets of measurement, these array data are nonetheless informative in providing insights into lncRNA function and regulation^13^.

We focused our study on the sponge regulation of those established and putative protein-coding driver genes in prostate cancer, which also showed expression variation across different disease states (Methods) and hence were likely to be functional^13^. By applying our integrated computational approach, we constructed a sponge regulatory network, in which each edge connects a potential sp-lncRNA to its corresponding PCGs. This network contains in total 96 predicted regulatory interactions between 52 sp-lncRNAs and 17 PCGs (Fig. 1c, Table 1 and Supplementary Data 1). Some PCGs such as PTEN and MLL2 (also known as KMT2D) showed greater numbers of predicted sp-lncRNAs than others (Table 1), suggesting that they might be subject to greater sponge regulation. Most PCGs in the network had more than one predicted sp-lncRNAs and many sp-lncRNAs regulated multiple PCGs, suggesting the existence of combinatorial regulation.

### The regulation of PTEN expression is 3′UTR-dependent

For experimental validation, we focused on sp-lncRNAs (Supplementary Data 2) of PTEN, which is among the protein-coding driver genes with the largest number of the predicted sp-lncRNAs in prostate cancer (Table 1). PTEN is a tumour suppressor that is one of the most frequently mutated protein-coding driver genes and often exhibits reduced expression in prostate cancer and many other cancers^14^. PTEN encodes a protein phosphatase, which can remove a phosphate from phosphoinositides at the plasma membrane^15^ and negatively regulates the PI3K/Akt pathway^16^,^17^. PTEN loss has been found in 9–45% of high-grade prostatic intraepithelial neoplasia, an abnormality of prostatic glands believed to precede the development of adenocarcinoma^18^,^19^,^20^,^21^. About 50–70% of castration-resistant prostate cancers (CRPCs) have genomic alterations in the PTEN/PI3K pathway, mostly through genetic loss of PTEN^22^,^23^,^24^. Loss of PTEN expression is associated with a more aggressive form of prostate cancer^14^,^25^,^26^. In the absence of genetic loss or mutation, PTEN can be downregulated in cancers by other mechanisms such as miRNA-mediated repression. Both pseudogene^12^ and the 3′ untranslated region (3′UTR) of PCG^27^,^28^ have been shown to influence PTEN expression through the sponge regulation mechanism.

**Figure 2:**
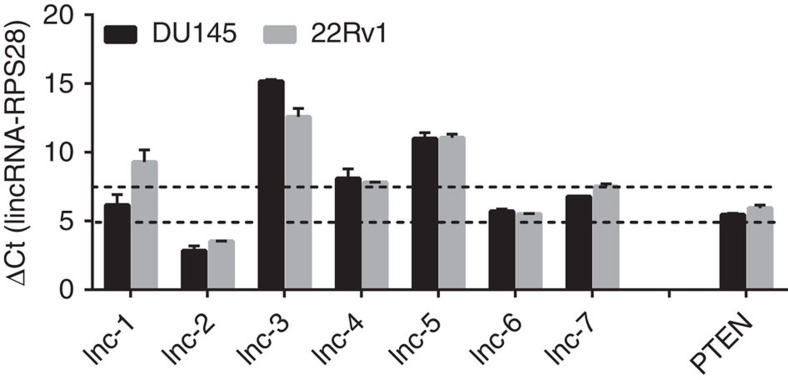
The relative expression levels of candidate sp-lncRNAs. The relative expression of seven candidate sp-lncRNAs of PTEN and PTEN in DU145 and 22RV1 cells was measured by real-time reverse transcription–PCR. The cycle threshold (Ct) difference (ΔCt) between a lncRNA gene and the reference gene RPS28 in the qRT–PCR experiment, which is inversely proportional to the amount of target nucleic acid in the sample, is shown. All experiments were performed in three replicates (*n*=3). Error bars are defined as s.d.

**Figure 3:**
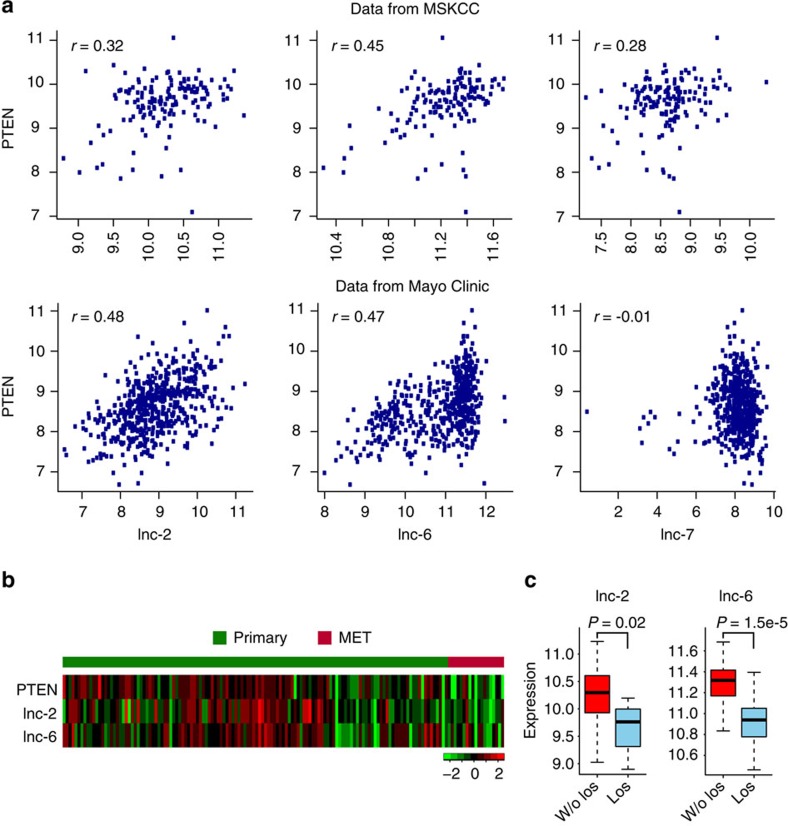
The genetic alteration and the expression profile of the predicted sp-lncRNAs. (**a**) The scatter plots show the correlation of expression between the predicted sp-lncRNAs and PTEN in both the MSKCC and the Mayo Clinic cohorts. (**b**) The heat map shows the expression variation of lnc-2, lnc-6 and PTEN across primary and CRPC tumours from the MSKCC cohort. (**c**) The Turkey boxplot shows the expression distribution of lnc-2 and lnc-6 in tumours with copy number loss and in the tumours without loss. The whiskers correspond to the lowest datum still within 1.5 interquartile range (IQR) of the lower quartile, and the highest datum still within 1.5 IQR of the upper quartile, respectively. Mann–Whitney *U*-test was performed for the comparison.

Among those sp-lncRNAs that were targeted by more than eight experimentally validated PTEN-regulating miRNAs, we chose two sp-lncRNAs lnc-2 (CTB-89H12.4, ENSG00000230551) and lnc-6 (Taurine Upregulated Gene 1 (TUG1), ENSG00000253352; Supplementary Data 3) that showed consistently the highest expression in two prostate cancer cell lines (DU145 and 22RV1) with wild-type PTEN for experimental validation (Fig. 2). We chose the higher expressed sp-lncRNAs because the higher expression makes a more effective sp-lncRNA given similar other conditions. Lnc-2 and lnc-6 showed a consistently positive correlation in expression with PTEN in the memorial sloan kettering cancer center (MSKCC)^24^ (*r*_lnc-2-PTEN_=0.32, *p*_lnc-2-PTEN_<5.89x10^−5^, *r*_lnc-6-PTEN_=0.45, *p*_lnc-6-PTEN_<5.89x10^−9^) cohort and Mayo Clinic^29^ (*r*_lnc-2-PTEN_=0.48, *p*_lnc-2-PTEN_<1.62x10^−32^, *r*_lnc-6-PTEN_=0.47, *p*_lnc-6-PTEN_<1.66x10^−31^) cohort (Fig. 3a). To assess the utility of using the co-expression data from these two cohorts instead of one cohort for predicting candidate sp-lncRNAs, we decided to test another lncRNA lnc-7 (ENSG00000267520) that shared 22 miRNAs with PTEN, but did not show consistent co-expression with PTEN in different cohorts (Fig. 3a and Supplementary Data 3 and 4). The genetic alternation and expression profile of lnc-2 and lnc-6 across normal prostate and prostate tumours suggested that they might exert a tumour-suppressive function in both primary prostate cancer and CRPC. First, their expression was decreased in CRPC tumours compared with primary tumours (Fig. 3b); second, lower expression was seen in tumours that harboured copy number loss (Fig. 3c).

**Figure 4:**
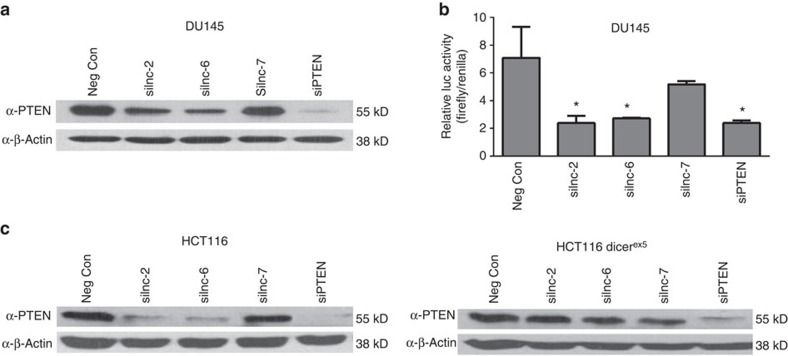
Experimental validation of the predicted PTEN regulation by sp-lncRNAs. (**a**) Western blot for PTEN protein level in DU145 cells transfected with the siRNA against lnc-2, lnc-6, lnc-7 and PTEN as well as the negative control (Neg Con) siRNA. (**b**) The bar graph shows the luciferase activity in DU145 cells co-transfected with a luciferase-PTEN-3′UTR reporter construct and the siRNA against lnc-2, lnc-6, lnc-7, PTEN as well as the Neg Con siRNA. (**c**) Western blot for PTEN protein level in wild-type HCT116 and HCT116 Dicer^ex5^ cells transfected with the siRNA against lnc-2, lnc-6, lnc-7 and PTEN as well as the Neg Con siRNA. All experiments with error bars were performed in three replicates (*n*=3). Error bars are defined as s.d. The two-sample *t*-test was used to calculate the significance of difference between the means of two experimental groups (*P<0.05, **P<0.01, NS: not significant, *P*⩾0.05).

To interrogate the function of these three sp-lncRNAs, we designed four independent short interfering RNAs (siRNAs) for each lncRNA genes and pooled those that showed efficient knockdown capability in the experiments (Methods). The effective siRNA-mediated knockdown of the candidate sp-lncRNAs was confirmed by quantitative real-time reverse-transcription PCR (qRT–PCR) analysis (Supplementary Fig. 1a). Consistent with the role of sp-lncRNAs as positive regulators of gene expression, the depletion of lnc-2 and lnc-6 transcripts by siRNAs in the DU145 prostate cancer cell line led to a significant reduction in PTEN expression (Fig. 4a). The effect on PTEN expression by siRNA-mediated silencing of either lncRNA was further confirmed in the 22Rv1 cell line (Supplementary Fig. 1b). Reciprocally, we found that depletion of PTEN transcript by siRNAs reduces the expression of lnc-2 and lnc-6, respectively (Supplementary Fig. 1c). The depletion of lnc-2 and lnc-6 transcripts also reduced the expression of two other PCGs, VAPA and SERINC1 (Supplementary Fig. 1d), which were previously shown to serve as sponge-mRNAs of PTEN^28^. With lower expression level than that of lnc-2 and lnc-6, other predicted sp-lncRNAs including lnc-1, 3, 4 and 5 showed a much weaker effect on PTEN expression (Supplementary Fig. 1d), indicating that the expression level is an important determinant of the efficacy of a miRNA sponge.

To further confirm the sponge regulation of PTEN by lnc-2 and lnc-6, we determined whether overexpressing lnc-2 or lnc-6 could rescue PTEN downregulation caused by miRNAs. Because of the large size of lnc-2 (ENST00000499521, 8,636 bps) and lnc-6 (ENST00000519077, 5,673 bps), we were only able to clone two sub-sequences of lnc-2 (703-4834 and 3931-8636) that contain the majority of the binding sites of the PTEN-regulating miRNAs into an expression vector (Methods), but not for the sub-sequences of lnc-6. Overexpressing either of the two lnc-2 sub-sequences (Supplementary Fig. 1e) rescued the downregulation of PTEN expression caused by overexpressing known PTEN-regulating miRNAs (Supplementary Fig.1f). In contrast to lnc-2 and lnc-6, the depletion of lnc-7 transcripts by siRNAs had no effect on PTEN expression (Fig. 4a), underscoring the importance of using co-expression data from multiple cohorts to ensure the robustness of the computational prediction.

Lnc-6 is officially known as TUG1, a highly conserved lncRNA expressed in the developing retina and brain as well as in adult tissues 30. TUG1 can be upregulated by p53 upon DNA damage in p53 wild-type, but not p53 mutant cells^3^,^31^. It associates with polycomb repressive complex 2 complex and represses the expression of cell-cycle genes^31^. In addition, TUG1 is involved in Polycomb 2 protein (Pc2)-mediated relocation of transcription units in the three-dimensional space of the nucleus^32^. Our discovery of TUG1 as a sp-lncRNA of PTEN established its cytoplasm function, which is consistent with previous studies showing an extensive localization of TUG1 in the cytoplasm^31^,^33^.

Next, we used a luciferase-PTEN-3′UTR reporter system to investigate whether the observed regulation of PTEN by lnc-2 or lnc-6 is via the PTEN 3′UTR. The use of this reporter assay allows for uncoupling the regulatory effect of sp-lncRNAs on PTEN through its 3′UTR from the effects through non-3′UTR mechanism such as PTEN transcription. We found that the siRNA-mediated knockdown of either lnc-2 or lnc-6 significantly reduced the chimeric luciferase reporter activity, whereas the knockdown of lnc-7 had little effect on the luciferase activity (Fig. 4b). These results suggest that the regulation of PTEN by sp-lncRNAs is through the PTEN 3′UTR.

### The regulation of PTEN expression is dependent on miRNAs

To further determine whether sp-lncRNA-mediated PTEN regulation is dependent on miRNA, we compared the difference of PTEN regulation by the candidate sp-lncRNAs in isogenic HCT116 colon cancer cell lines. The only difference between the two isogenic cell lines is that one has a wild-type DICER, whereas the other has a mutant DICER (DICER^ex5^) with an insertion disruption in the N-terminal helicase domain. This hypomorphic mutation in DICER impaired its function in the maturation of the vast majority of miRNAs^34^. It has been shown^28^ that the levels of mature PTEN-regulating miRNAs in the HCT116 DICER^ex5^ cell line are significantly decreased, whereas the siRNA-mediated silencing is fully functional. Therefore, the DICER^Ex5^ cell line serves as an ideal system to evaluate the miRNA dependency of sp-lncRNA-mediated PTEN regulation. Similar results were observed in DU145 and 22Rv1 cell lines, where the depletion of lnc-2 or lnc-6 by siRNAs substantially reduced PTEN expression, whereas the depletion of lnc-7 had no effect on PTEN expression in the wild-type HCT116 (Fig. 4c). In contrast, in the DICER^ex5^ cell line, the downregulation of PTEN by the loss of lnc-2 or lnc-6 was considerably impaired (Fig. 4c). These results suggest that the sp-lncRNA-mediated PTEN regulation is critically dependent on the Dicer-mediated miRNA activity.

### The determinants of sponge lncRNA efficacy

**Figure 5:**
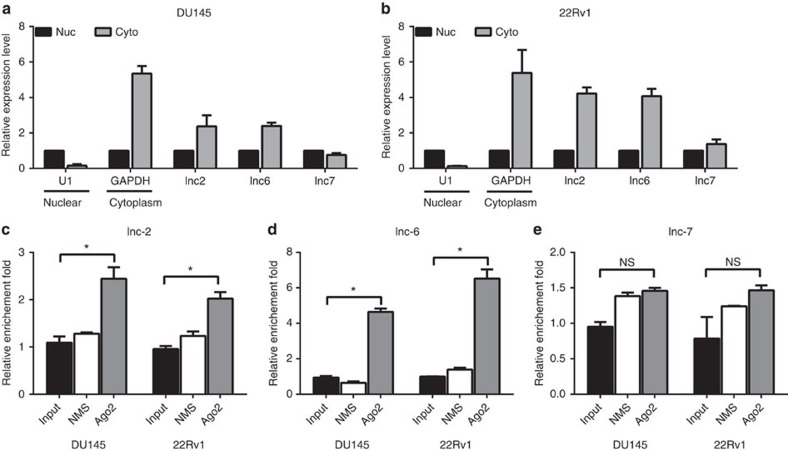
The sub-cellular localization and the RISC accessibility of lncRNAs. The RNA level of lnc-2, lnc-6 and lnc-7 in nuclear (Nuc) and cytoplasmic (Cyto) fraction was determined by RT–PCR in (**a**) DU145 and (**b**) 22Rv1 cells, respectively. U1 was a positive control for Nuc fraction and GAPDH was a positive control for Cyto fraction. Anti-Ago2-RIP-ChIP for (**c**) lnc-2, (**d**) lnc-6 and (**e**) lnc-7 in DU145 and 22Rv1 cell lines. The relative enrichment with respective to total RNA (input) in both anti-Ago2-RIP and Nonspecific Mouse Serum (NMS) control are shown. All experiments were performed in three biological replicates (*n*=3). Error bars are defined as s.d. The two-sample *t*-test was used to calculate the significance of difference between the means of two experimental groups (*P<0.05, **P<0.01, NS: not significant, *P*⩾0.05).

Although lnc-7 was predicted to share 22 miRNAs with PTEN, it had no regulatory effect on PTEN expression. We further investigated the mechanism, whereby lnc-7 was unable to serve as an effective miRNA sponge. The miRNA-induced repression occurs dominantly in the cytoplasm and is mediated by RISC. We thus hypothesized the reason why lnc-7 cannot serve as an effective sponge is because it is not predominantly localized in the cytoplasm and is not effectively accessible to the RISC. To test this hypothesis, we performed subcellular fractionation followed by qRT–PCR (Fig. 5a,b) to examine the subcellular localization of in the DU145 cell line. Indeed, lnc-2 and lnc-6 were predominantly localized in the cytoplasm in the DU145 and 22Rv1 cell lines, whereas lnc-7 was not (Fig. 5a,b).

To further confirm the subcellular localization of the lnc-2, lnc-6 and lnc-7, we employed a single-molecule RNA fluorescence *in situ* hybridization (RNA-FISH) method as previously described^35^,^36^. We used the Biosearch probe design algorithm (Biosearch Technologies, Inc.) to make the probes for the lncRNAs and targeted the exons of each lncRNA using probes conjugated to Quasar 570 fluorophore (Methods). The specificity of the probe sets was validated as previously described^36^,^37^. Briefly, we partitioned each probe set to the even- and odd-numbered oligonucleotides and coupled each subset with a different fluorophore (evens with Quasar 570 fluorophore, odds with Quasar 670 fluorophore). We then hybridized the two probe sets and imaged each channel, separately. If a probe set is specific to the lncRNA of interest, one would expect that the signal from even and odd probe sub-set would show good co-localization. Because the specificity of the probe set for lnc-6 was validated in a previous study^38^, herein we focused on validating the specificity of probe sets for lnc-2 and lnc-7. We found the even (red) and odd (green) probe set signal showed good co-localization for both lnc-2 and lnc-7 (Supplementary Fig. 2), indicating a good specificity of these probe sets. Our RNA-FISH analysis revealed a predominantly cytoplasmic distribution for the lnc-2 and lnc-6 in DU145 (Supplementary Fig. 3a,b) and 22Rv1 (Supplementary Fig. 3d,e) cell lines, but not for lnc-7 (Supplementary Fig. 3c,f), in concordance with our biochemical fractionation experiments. Therefore, both the lower expression and the lower cytoplasmic localization of lnc-7, in comparison with lnc-2 and lnc-6, reduced its efficacy as a miRNA sponge.

To ascertain the accessibility of the Ago-containing RISC to lnc-2, lnc-6 and lnc-7, we performed anti-Ago2-ribonucleoprotein immunoprecipitation (RIP) followed by array hybridization (RIP-ChIP) experiments. The RIP-ChIP found that compared with a nonspecific mouse serum (NMS) control, lnc-2 and lnc-6 were significantly enriched in the anti-Ago2-RIP fraction in both DU145 and 22Rv1 cell lines (Fig. 5c,d), whereas lnc-7 was not (Fig. 5e). Therefore, although lnc-7 sequence harbours potential miRNA-binding sites, it was not effectively accessible to the RISC for miRNA targeting and was unable to serve as an effective miRNA sponge.

### Sponge lncRNAs of PTEN exert a tumour-suppressive function

**Figure 6:**
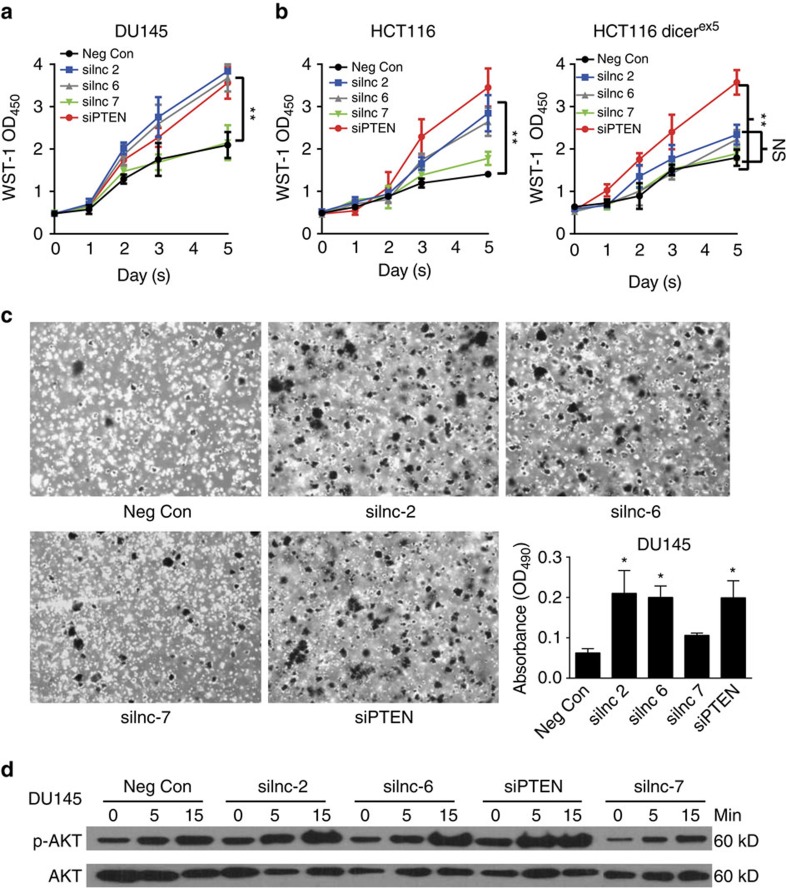
Functional validation of the predicted sp-lncRNAs of PTEN. (**a**) Cell proliferation curve of DU145 cells transfected with the siRNA against lnc-2, lnc-6, lnc-7 and PTEN as well as the negative control (Neg Con) siRNA. (**b**) Cell proliferation curves of the HCT116 Dicer wild-type and HCT116 Dicer^ex5^ cells transfected with the siRNA against lnc-2, lnc-6, lnc-7 and PTEN as well as the Neg Con siRNA. (**c**) Anchorage-independent growth of DU145 cells transfected with the siRNA against lnc-2, lnc-6, lnc-7 and PTEN as well as the Neg Con siRNA. The bar graph shows the quantification of the colony formation after 10 days. (**d**) Western blot for phospho-AKT and AKT level following serum starvation and restimulation of DU145 cells transfected with the siRNA against lnc-2, lnc-6, lnc-7 and PTEN as well as the Neg Con siRNA. All experiments with error bars were performed in three replicates (*n*=3). Error bars are defined as s.d. The two-sample *t*-test was used to calculate the significance of difference between the means of two experimental groups (*P<0.05, **P<0.01, NS: not significant, *P*⩾0.05).

PTEN serves as a tumour suppressor to negatively regulate cancer cell growth or survival by reducing the activity of the oncogenic PI3/Akt pathway^16^,^17^. We therefore tried to determine, as the positive regulators of PTEN expression, whether the sp-lncRNAs of PTEN also exert a tumour-suppressive function. In the prostate cell line DU145, the reduction of either lnc-2 or lnc-6 expression by siRNA significantly increased cell proliferation, which partially phenocopied the effect of siRNA-mediated silencing of PTEN (Fig. 6a). This growth promotion upon sp-lncRNA knockdown was further confirmed in the 22Rv1 cell line (Supplementary Fig. 4a), suggesting that both lnc-2 and lnc-6 exerted a tumour-suppressive function. Consistent with the observation that lnc-7 depletion had no effect on PTEN expression, its depletion had no effect on prostate cancer cell proliferation (Fig. 6a and Supplementary Fig. 4a). The effect on cell proliferation upon siRNA-mediated silencing of lnc-2 and lnc-6 was similar in the wild-type HCT116 cells compared with that in DU145 cells, but was considerably dampened in the HCT116 DICER^ex5^ cell line (Fig. 6b). The difference between wild-type and DICER^ex5^ HCT116 cells further supports that the tumour-suppressive function of PTEN sp-lncRNAs is miRNA dependent. Moreover, the siRNA-mediated depletion of either lnc-2 or lnc-6 but not of lnc-7 significantly increased anchorage-independent cell growth from soft-agar colony formation assay (Methods) in DU145 (Fig. 6c) and 22RV1 (Supplementary Fig. 4b) cells. The reduction of either lnc-2 or lnc-6 expression by siRNA also significantly increased anchorage-independent cell growth of the wild-type HCT116 cells (Supplementary Fig. 4c), but the effect was considerably reduced in the DICER^ex5^ HCT116 cell line (Supplementary Fig. 4d).

The tumour-promoting effect of PTEN loss/reduction in human cancer can be partially attributed to an aberrant elevation of the PI3K/Akt pathway activity^14^,^16^,^17^. To further determine the molecular underpinning of the tumour-suppressive function of PTEN sp-lncRNAs, we assessed the impact of PTEN-regulating sp-lncRNA on the PI3K/Akt pathway activity. Indeed, the siRNA-mediated silencing of the lnc-2 and lnc-6 significantly elevated phospho-Akt levels in response to serum stimulation (Fig. 6d).

## Discussion

LncRNAs have recently emerged as natural miRNA sponges, which play important roles in various biological processes such as muscle differentiation (linc-MD1 (ref. 10)) and embryonic stem cell self-renewal (lincRNA-RoR^39^,^40^). By integrating gene expression profile data of both lncRNAs and PCGs in tumours and the sequence features of RNAs, we uncovered a lncRNA-mediated sponge regulatory network of protein-coding driver gene expression in prostate cancer. We revealed that the sponge regulation by lncRNA had a widespread influence on the expression of key components of the cancer-driving circuits and those sp-lncRNAs may themselves serve as oncogenes or tumour suppressors. Furthermore, the regulation of a protein-coding driver gene expression by sp-lncRNAs was not a simple one-to-one, but a many-to-many relationship: individual protein-coding driver genes were regulated by multiple sp-lncRNAs and one sp-lncRNAs could regulate many protein-coding driver genes.

The regulatory function of two computationally predicted sp-lncRNAs of PTEN, a master tumour suppressor in prostate cancer was experimentally confirmed. These two lncRNAs not only regulated PTEN expression in a miRNA-dependent manner, but also demonstrated tumour-suppressor activities in prostate cancer cell lines. Moreover, both lncRNAs exhibited concordance between expression reduction and copy number loss in prostate cancer, representing strong genetic evidence of their function *in vivo*. In CRPC, both lncRNAs were downregulated compared with primary prostate tumours, suggesting that they might have an important function in advanced prostate cancer by downregulating PTEN expression level.

One of the validated PTEN sp-lncRNA lnc-6 (TUG1) was previously known to be involved in polycomb repressive complex 2-mediated transcriptional regulation and the three-dimensional organization of the transcription unit in the nucleus. The newly discovered cytoplasm function TUG1, a miRNA sponge, indicates that a lncRNA can have multiple functions depending on its sub-cellular localization. This underappreciated functional plasticity of individual lncRNA could form the basis for their context-dependent function. Moreover, we showed that the expression level, the cytoplasmic localization and/or the accessibility to the RISC are important factors for determining the sponge efficacy of a lncRNA.

In summary, our study reveals a prevalent and complex lncRNA-mediated sponge regulatory mechanism that may significantly contribute to the aberrant expression of critical protein-coding driver genes in prostate cancer. Those sp-lncRNAs might have oncogenic or tumour-suppressive function and perturbation of the lncRNA-mediated sponge regulation might be exploited for cancer therapy. Our study also suggests the vast functional space of lncRNAs as miRNA sponges in cancer pathogenesis and the enormous plasticity of lncRNAs in performing multiple functions.

## Methods

### Cell cultures

DMEM, RPMI-1640, McCoy's 5A and fetal bovine serum (FBS) are from Invitrogen. DU145, 22Rv1, HCT116 Dicer wild-type or HCT116 Dicer^ex5^ cells were grown in RPMI-1640 with 10% FBS, DMEM with 10% FBS or McCoy's 5A with 10% FBS, respectively. 22Rv1 and DU145 cell lines were obtained from American Type Culture Collection, HCT116 Dicer wild-type and Dicer^ex5^ cells were a kind gift from Dr Vogelstein's group from the Johns Hopkins University School of Medicine. All cell lines were authenticated using Promega Power Plex 16HS Kit (Promega Inc.) and were tested to ensure no mycoplasma contamination using MycoSEQ Mycoplasma detection kits (Thermo Fisher Scientific Inc.). All cell lines were grown in penicillin/streptomycin and glutamine containing medium, at 37 °C in a humidified atmosphere with 5% CO_2_.

### Transient transfection

SiGENOME non-targeting siRNA #2 (siLuc), siPTEN smartpool and all siRNAs for lncRNAs are from Dharmacon. The sequences of siRNAs used to knockdown each candidate PTEN sp-lncRNA are listed in Supplementary Table 1. For the transfection of siRNAs, DU145 (3 × 10^5^) or 22Rv1 (2 × 10^5^) were seeded into six-well dishes. The following day they were transfected with 100 nM siRNAs using lipofectamine^2000^ (Invitrogen Inc.) according to the manufacturer's recommendations. PTEN 3'UTR overexpression was achieved by transient transfection using pGL3luc expression vectors. MiRNA/target interaction was measured by a luciferase reporter assay. PTEN and lncRNA expression levels were detected by qRT–PCR.

### Dual luciferase reporter assay

DU145, 22Rv1, HCT116 Dicer wild-type or HCT116 Dicer^ex5^ cells were seeded at a density of 2 × 10^5^ cells per six-well dish. Twenty-four hpurs later, 1,000 ng of pGLU/PTEN-3′UTR were co-transfected with 100 ng of pRL-TK using Lipofectamine^2000^. Forty-eight hours after transfection, the luciferase activity was measured by Dual-Luciferase reporter assay kit and normalized. PGL3-control, pRL-TK and Dual-Luciferase reporter assay kit are from Promega.

### RNA extraction and qRT–PCR

For qRT–PCR analyses, total RNA was extracted from cells using Trizol reagent (Invitrogen Inc.) as per the manufacturer's instructions and subsequently column purified with RNeasy kits (Qiagen). cDNA synthesis was performed using the High-Capacity cDNA Archive kit (Applied Biosystem) and SuperScript II reverse transcriptase (Invitrogen Inc.) according to the manufacturer's instructions. The qRT–PCR primer sequences are listed in the supplementary Data 5.

### Western blot analysis

Cells were collected and lysed (50 mM Tris, pH 8.0, 1 mM EDTA, 1 mM MgCl_2_, 150 mM NaCl, 1% NP-40, 1 mM β-glycerophosphate, 1 mM Na_3_VO_4_, 1 mM NaF, protease inhibitors). Proteins (30 μg per lane) were separated on 10% SDS–polyacrylamide gel and transferred to nitrocellulose membrane. Immunoblotting of the membranes was performed using the following primary antibodies: anti-PTEN (1:2,000), anti-AKT (1:2,000), anti-Phospho-AKT(1:2,000) or anti-β-actin (1:5,000). Anti-PTEN (#9559), anti-AKT (#9272) and anti-Phospho-AKT Ser473 (#9271) are from Cell Signaling; anti-β-actin antibody is from Sigma-Aldrich. Signals were revealed after incubation with recommended secondary antibody coupled to peroxidase by using enhanced chemiluminescence. Scanned images were quantified using ImageJ software. The uncropped scans of the most important western blots are shown in the Supplementary Figure 5.

### Overexpression of lncRNAs and PTEN-regulating miRNAs

Lnc-2 fragment 1 (F1, ENST00000499521, 703-4834) and fragment 2 (F2, ENST00000499521, 3931-8636) were successfully cloned into an EF-1 alpha-promotor-driven expressing vector. For PTEN expression rescue experiment, transfection reagent only (Mock), miRs (3 nM each of miR-106a, -106b, -17-5p, -19a, -19b, -20a, -20b, 26a, -26b and -93), negative miR control (30 nM), empty expression vector (100 ng, vector control), fragment 1 (100 ng, F1), fragment 2 (100 ng, F2) were transfected separately or co-transfected together (F1+miRs, F2+miRs) into DU145 cells. Synthetic, chemically modified short single-stranded RNA oligonucleotides: Pre-miR-106a, Pre-miR-106b, Pre-miR-17-5p, Pre-miR-19a, Pre-miR-19b, Pre-miR-20a, Pre-miR-20b, Pre-miR-26a, Pre-miR-26b, Pre-miR-93 and Pre-miR-negative control,were purchased from Ambion/Life Technologies. All TaqMan primers and probes were purchased from Applied Biosystem. Forty-eight hours after transfection, cells were collected and expression levels of miRs, lnc-2 or PTEN were detected by qRT–PCR or western blot analysis.

### Nucleus-cytoplasm fractionation

Both nuclear and cytoplasmic RNA from cultured DU145 or 22Rv1 cells were isolated by SurePrep Nuclear or Cytoplasmic RNA Purification Kit (Fisher Scientific BP2805-25) followed manufactuere's instruction. U1 RNA and GAPDH processed mRNA were detected in isolated RNAs as control for nuclear RNA and cytoplasm RNA, respectively. Biological triplicates were carried out and followed by qRT–PCR to detect abundance of lncRNAs.

### RNA-FISH

The FISH protocol was performed as described previously^36^. The oligonucleotide probes were designed and purchased from Biosearch Technologies, Inc. The sequences of RNA-FISH probes are listed in Supplementary Data 6. The probes targeting lncRNA exons were conjugated to the fluorophore Quasar 570. Briefly, before fixation and hybridization, DU145 and 22Rv1 cells were plated on the two-chamber dishes (Nunc Lab-Tek Chambered Coverglass, Thermoscientific cat. no. 155380) and grown overnight at 37 °C. Next day, the cells were fixed in 1 ml of 4% formaldehyde for 10 min at room temperature, washed with 1 × PBS twice and permeabilized with 70% EtOH in two-chamber dishes. Before the hybridization, the cells were rehydrated with wash buffer containing 10% formamide (Ambion, cat. no. AM9342) and 2 × sodium citrate buffer (SSC; Ambion, cat. no. AM9765) for 5 min. Then, the probes (0.3–0.6 μM final) were hybridized in 10% dextran sulfate (Sigma, cat. no. D8906), 10% formamide and 2 × SSC at 37 °C overnight. After hybridization, the cells were washed in wash buffer at 37 °C for 30 min twice (with the addition of 4,6-diamidino-2-phenylindole (1 × ) in the second wash) and then in 2 × SSC twice. The imaging was done immediately after with 2 × SSC as the mounting medium. More than 70 nuclei for each lncRNA across multiple passages were examined. 4,6-Diamidino-2-phenylindole and Cy3 channels were used to detect the nuclei and the exon signals, respectively. To further check for background autofluorescence, the FITC channel was used for imaging, and it was confirmed that the exon signals did not co-localize with the signals observed in the FITC channel. Across all samples, 27–33 z-stacks, each 0.33 μm, were taken.

### Ago RIP-ChIP

Ago RIP-ChIP was performed as previously described^41^. The monoclonal antibody against Ago2 was purchased from Abcam (ab57113). Briefly, DU145 or 22Rv1 cells were rinsed and lysed on ice with fresh lysis buffer with protease inhibitors. Cell lysates were collected and cleared with pre-blocked Protein G beads (Invitrogen), and proceeded for co-IP with either anti-AGO G beads, Nonspecific Mouse Serum (NMS, Pierce Biotechnology) G beads at 4 °C for 90 min. RNAs that co-IP with anti-AGO antibodies were extracted using TRIzol (Invitrogen). Biological triplicates were carried out and followed by qRT–PCR detection for the enrichement of lncRNAs. We used the anti-AGO-RIP-ChIP approach, which uses anti-AGO to IP AGO-containing miRNPs and associated mRNAs. MiRNPs in total cell lysates from DU145 and 22Rv1 cell lines were co-IP with anti-AGO and investigated. As controls, IP with a non-immune serum were performed in parallel.

### Cell proliferation

Eight hours after transfection, 1 × 10^5^ DU145, 22Rv1, HCT116 Dicer wild-type or HCT116 Dicer^ex5^ cells were trypsinized, resuspended in 50 ml and seeded in 8 sets of 4 wells of a 96-well plate. Starting from the following day (d0), 1 set of wells per day was washed once with PBS, stained with WST-1 (Roche) at 450 nm according to the manufacturer's recommendation until day 5.

### Growth in semisolid medium

Anchorage-independent growth of DU145 and 22Rv1 cells transfected with siRNAs against PTEN sp-ncRNAs, siRNA-negative control or siPTEN were determined by using Cell Transformation Detection Assay Kit (Millipore) according to the manufacturer's instructions. Briefly, the bottom layer was obtained by covering six-well dishes with 2 ml of 0.6% agar in DMEM. The 10 days after, 5 × 10^4^ transected cells were seeded on top in triplicate in 2 ml of 0.3% agar in DMEM+10% FBS. Colonies were counted after 3–4 weeks at × 40 magnification.

### Statistical analysis of experimental results

*In vitro* data were analysed using unpaired *t*-test (GraphPad Prism, GraphPad Software, Inc.) Values of *P*<0.05 were considered statistically significant (*). The mean±s.d. of three or more independent experiments is reported. Regression analyses and correlation coefficients were generated using GraphPad Prism, GraphPad Software, Inc.

### Genomic and clinical data of prostate cancer

Two sets of exon array data of prostate cancer, which were generated by MSKCC Prostate Oncogenome Project^24^ and by Mayo Clinic^29^, respectively, were downloaded from Gene expression Omnibus (GEO; GSE21034 and GSE46691). In addition to the exon-array data, the MSKCC data set contains the matched clinical information and SCNA data.

### LncRNA expression from Affymetrix Human Exon 1.0 ST array

We collected lncRNA annotation from two sources: the catalogue of lncRNA from Ensembl database^42^ (Homo sapiens GRCh37, release 72) and the catalogue of lncRNA generated based on transcriptome assembly from RNA-seq data^2^. For those lncRNA transcripts that have overlap on the same strand between these two sources, we only kept the Ensembl annotation to avoid redundancy. The re-annotation of the exon-array probes for interrogating lncRNA expression was performed as described previously^13^. A total of 11,271 lncRNA genes had at least four exon-array probes that were uniquely mapped uniquely to their exons. The gene expression summarization and normalization based on probe-level data were performed as described in our previous study^13^. We further performed coding potential analysis and 10,640 lncRNAs eventually were considered as noncoding and were selected for further study.

### Coding potential analysis

To confirm the lncRNA genes are noncoding as are annotated, we used the algorithm CPAT (http://lilab.research.bcm.edu/cpat) with the default parameter. CPAT is an alignment-free method^43^, which showed favourable performance compared with other coding potential prediction methods. For lncRNA gene with more than one transcript, we only considered it as noncoding if all of its transcripts were noncoding.

### MiRNA target prediction

MiRNA sequences and family information were obtained from TargetScan website (http://www.targetscan.org/). We selected miRNAs from conserved miRNA families and showed top 50% expression in the prostate cell line DU145. The target sites of miRNAs were predicted by using TargetScan^44^. For lncRNA transcripts, the default parameters were used to predict miRNAs' target sites. For PCGs, the predicted target sites on 3′UTRs were obtained from TargetScan website, in which 23-way alignment information was used for prediction.

### Predicting the sp-lncRNAs of protein-coding driver genes

We obtained a comprehensive list of protein-coding cancer driver genes, both oncogenes and tumour suppressors, from Vogelstein *et. al*.^45^. We focused on 45 driver genes in prostate cancer, which showed differential expression (two-tailed Mann–Whitney *U* test, *P*≤0.01) between normal prostate and primary tumours or between primary and metastatic tumours for further study. We devised a computational strategy to identify candidate sp-lnRNA-driver gene pairs (Fig. 1b). First, for each lncRNA-driver gene pair, we estimated the significance of shared miRNAs with the same seeds (*P*-value of one-tailed Fisher's exact test) and the significance of expression correlation across tumours in MSKCC data set (*P*-value of one-tailed Pearson's correlation coefficient test). We then computed a combined *P*-value by converting *P*-values of these two tests *P*_1_ and *P*_2_ by using the formula 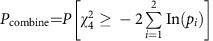, which is known as Fisher's method. The candidate sp-RNA-driver gene pairs met the criterion that the adjusted combined *P*-value is no larger than a threshold of 5% (that is, false discovery rate (FDR) ≤0.05). Second, we selected sp-RNA-driver gene pairs that shared at least ten different miRNAs and at least eight unique targeting sites for these shared miRNAs (different miRNAs sharing the same seed sequence could target the same site). Third, we selected the sp-RNA-driver gene pairs that showed at least moderate positive correlation of expression (*r*⩾0.25) in two independent clinical cohorts from MSKCC^24^ and Mayo clinic^29^.

## Additional information

**How to cite this article**: Du, Z. *et al*. Integrative analyses reveal a long noncoding RNA-mediated sponge regulatory network in prostate cancer. *Nat. Commun.* 7:10982 doi: 10.1038/ncomms10982 (2016).

## Supplementary Material

Supplementary Information Supplementary Figures 1-5 and Supplementary Table 1

Supplementary Data 1 The predicted sponge lncRNA and protein-coding gene pairwise regulations and their shared miRNAs/binding sites and correlation of expression in MSKCC and Mayo Clinic cohorts.

Supplementary Data 2 The predicted PTEN sponge lncRNAs and their genomic coordinates, ensemble ID, gene symbol, correlation of expression and shared miRNAs/binding sites with PTEN.

Supplementary Data 3 The predicted lncRNA-targeting of three candidate PTEN sponge lncRNAs (lnc2, 6 and 7) and their miRNA ID and the location information of miRNAs binding sites within the transcripts.

Supplementary Data 4 The ID, sequence, conservation and the cell-line expression level of the predicted PTEN-targeting miRNAs.

Supplementary Data 5 The qRT-PCR primer sequences of PTEN, rps28 and seven PTEN sponge lncRNA candidates (lnc1 to 7).

Supplementary Data 6 The sequences of RNA-FISH probes for three PTEN sponge lncRNA candidates (lnc2, 6 and 7).

## Figures and Tables

**Table 1:** The number of predicted sp-lncRNAs for protein-coding driver genes.

**Gene symbol**	**Number**
ARID2	7
BCL2	4
CBL	3
CCND1	1
CDC73	4
CYLD	6
DNMT3A	1
FAM123B	6
GATA2	1
KLF4	2
MLL2	13
PDGFRA	9
PIK3R1	7
PTEN	12
RUNX1	3
SMAD4	8
TET2	9

sp-lncRNA, sponge-long noncoding RNA.
