# Supplementary material for: Integrative analyses reveal a long noncoding RNA-mediated sponge regulatory network in prostate cancer
Source: Nat Commun. 2016 Mar 15;7:10982. doi: 10.1038/ncomms10982 (PMC4796315; doi:10.1038/ncomms10982)
Supplement: Supplementary Information — Supplementary Figures 1-5 and Supplementary Table 1 [file ncomms10982-s1.pdf]

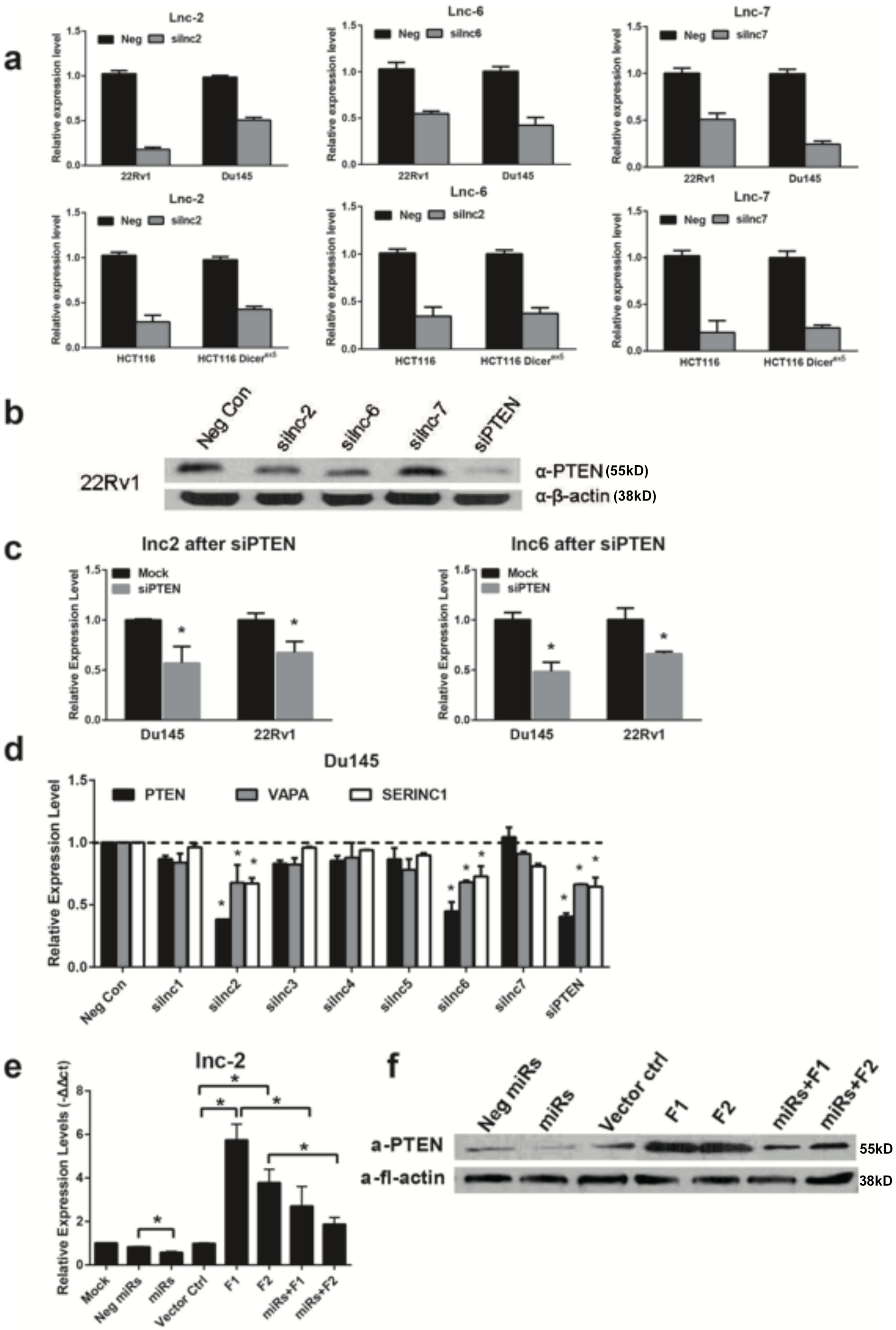

Supplementary Figure 1. (a) The qRT-PCR for lnc-2, lnc-6 and lnc-7 RNA level in DU145, 22Rv1, wild type HCT116 and HCT116 Dicer^{ex5} cells transfected with the siRNA against lnc-2, lnc-6, lnc-7, and the negative control siRNA. (b) Western blot for PTEN protein level in 22Rv1 cells transfected with the siRNA against lnc-2, lnc-6, lnc-7, and

PTEN as well as the negative control siRNA. **(c)** The qRT-PCR for lnc-2 and lnc-6 and RNA level in DU145 and 22Rv1 cells transfected with the siRNA against PTEN and the negative control siRNA. **(d)** The qRT-PCR for PTEN, VAPA and SERINC1 RNA level in DU145 cells transfected with the siRNA against PTEN, lnc1-7, and the negative control siRNA. **(e)** The relative expression level of lnc-2 (ΔΔct value normalized to mock control group) and (f) western blot for PTEN and actin control protein level in DU145 cells for the rescue experiment. Mock: tranfection agent only; Neg miRs: transfected with scrambled miR control; miRs: transfected with 10 PTEN-targeting miRs' precursors; Vector Ctrl: transfected with empty expressing vector only; F1: transfected with the expressing vector inserted with lnc-2 gene fragment 1; F2: transfected with the expressing vector inserted with lnc-2 gene fragment 2; miRs+F1: co-transfected with PTEN-targeting miRs' precursors and with the expressing vector inserted with lnc-2 gene fragment 1; miR+F2: co-transfected with PTEN-targeting miRs' precursors and with the expressing vector inserted with lnc-2 gene fragment 2. All experiments with error bars were performed in three replicates (n=3). Error bars are defined as SD. The two-sample t-test was used to calculate the significance of difference between the means of two experimental groups (*$p<0.05$, ** $p<0.01$).

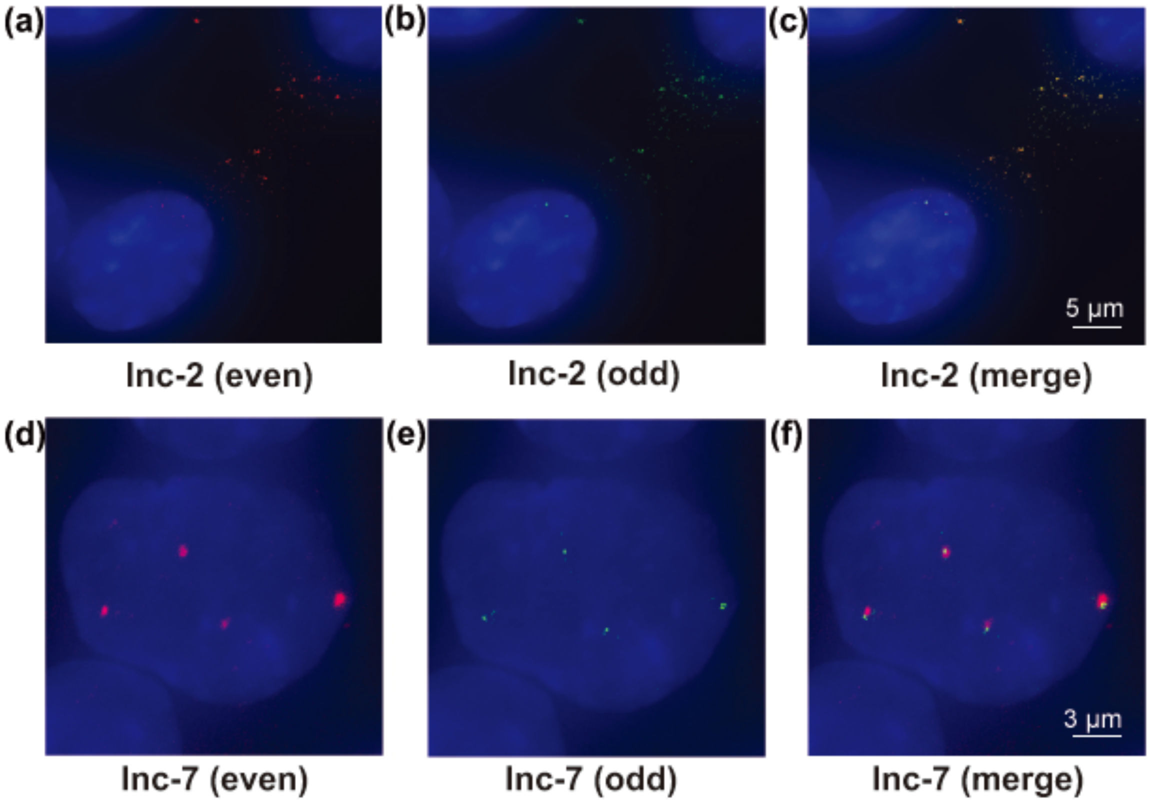

Supplementary Figure 2. RNA-FISH for probe set quality control (a) even probes of lnc-2, (b) odd probes of lnc-2, and (c) the merged image of (a) and (b) in DU145 cells, as well as (d) even probes of lnc-7, (e) odd probes of lnc-7 and (f) the merged image of (d) and (e). DAPI staining of nucleus is shown in blue. The FISH signal of even probes is shown in red and that of odd probes is shown in green.

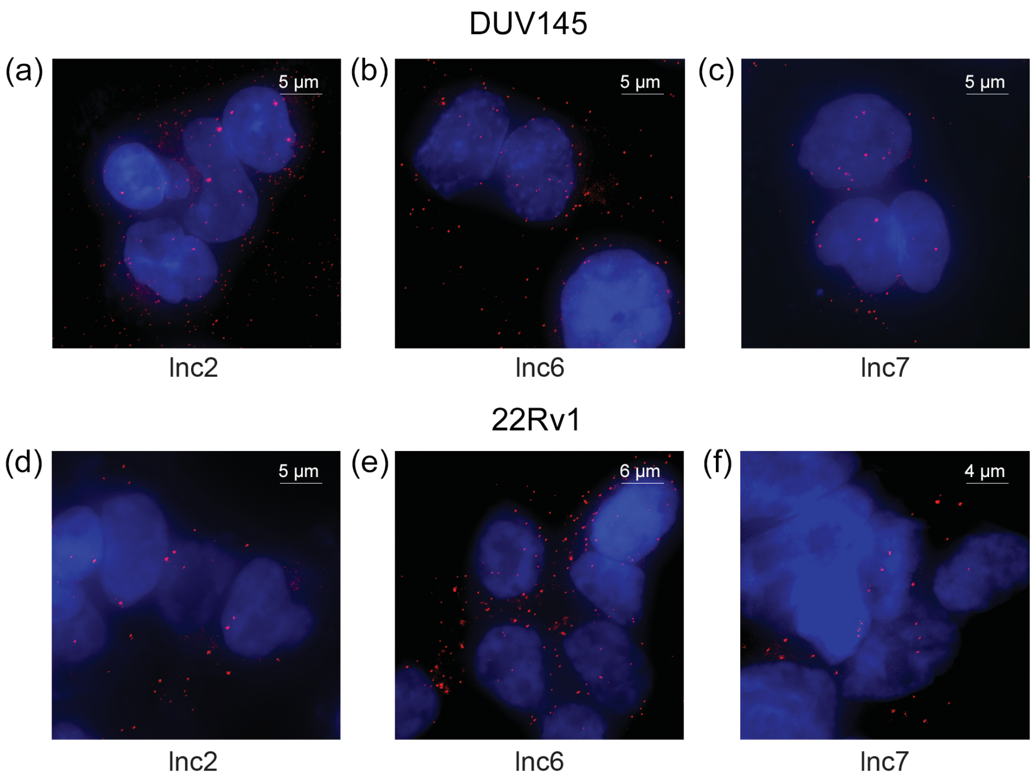

Supplementary Figure 3. RNA-FISH for determining subcellular localization of (a) lnc-2, (b) ln-6, and (c) lnc-7 in DU145 cells, as well as (d) lnc-2, (e) ln-6 and (f) lnc-7 in 22Rv1 cells. DAPI staining of nucleus is shown in blue and FISH signal is shown in red.

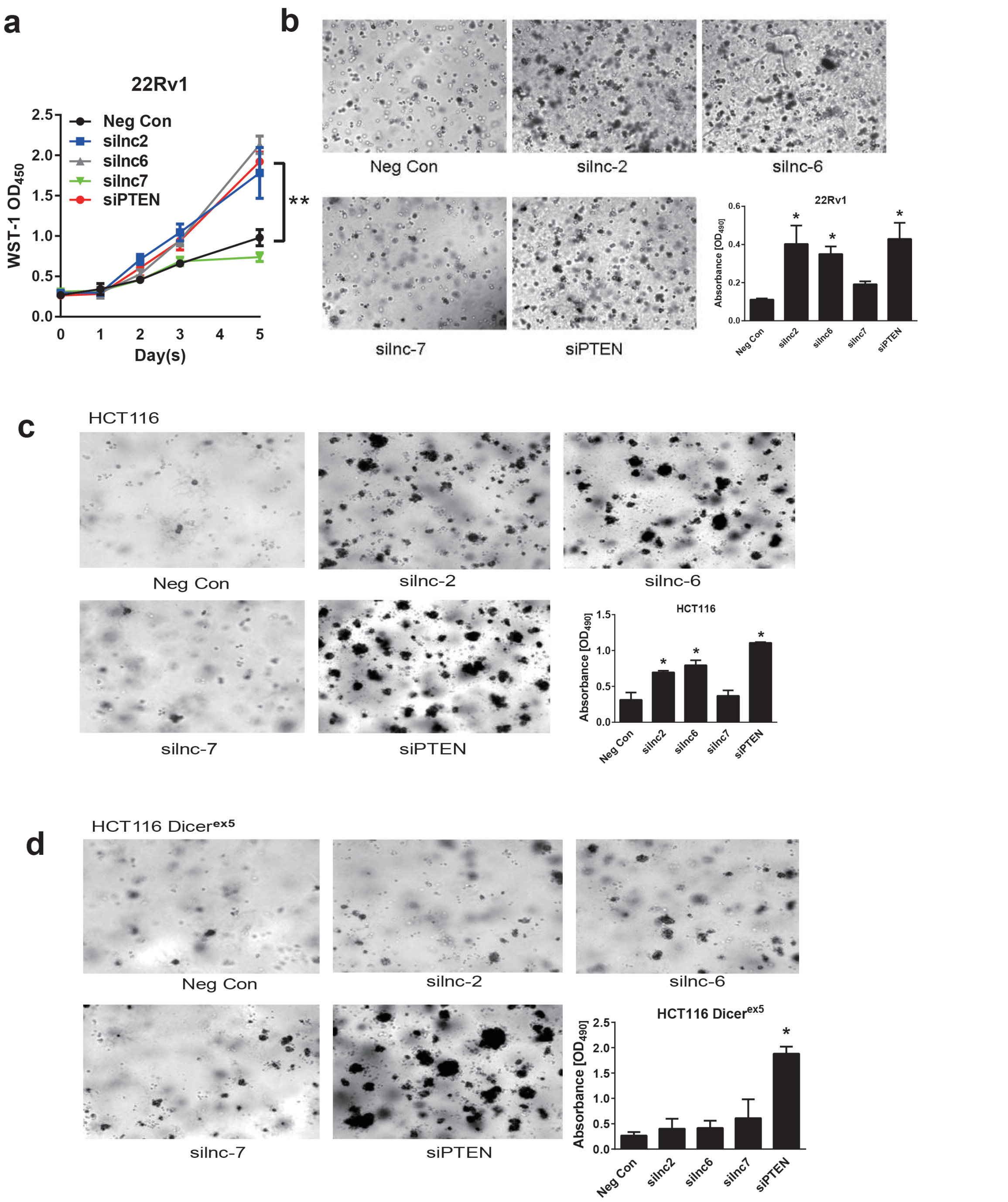

Supplementary Figure 4. (a) Cell proliferation curves of 22Rv1 cells transfected with the siRNA against lnc-2, lnc-6, lnc-7, and PTEN as well as the negative control siRNA. Anchorage-independent growth of (b) 22Rv1, (c) the wild type HCT116, and (d) the HCT116 DICER^{ex5} cells transfected with the siRNA against lnc-2, lnc-6, lnc-7, and PTEN as well as the negative control siRNA. All the bar graph showed the quantification of the colony formation after 10 days. All experiments with error bars were performed in three replicates (n=3). Error bars are defined as SD. The two-sample t-test was used to calculate the significance of difference between the means of two experimental groups and the one-way analysis of variance (ANOVA) was used to calculate the significance of difference between the means of more than two groups (*p<0.05, ** p<0.01).

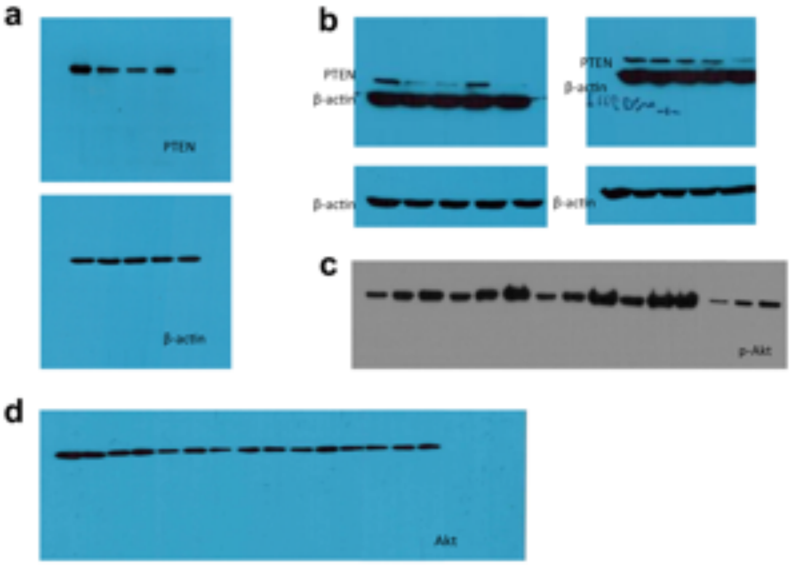

Supplementary Figure 5. The uncropped scans of (a) Western blot for PTEN protein level in DU145 cells (corresponding to Figure 4a), (b) Western blot for PTEN protein level in wild type HCT116 and HCT116 Dicer^{ex5} cells (corresponding to Figure 4c), (c) Western blot for phospho-AKT level following serum starvation and restimulation of DU145 cells (corresponding to Figure 6d), and (d) Western blot for AKT level following serum starvation and restimulation of DU145 cells (corresponding to Figure 6d).

lnc-2	
RNAi-1	UGUGUCUUCAACUUGUAUC
RNAi-3	UAUUACGGUACUAAUAUCCCU
RNAi-4	AUCCGCAUUUCCUGUUGGGUU
lnc-6 (TUG1)	
RNAi-1	GGATATAGCCAGAGAACAA
RNAi-3	GAATAAGCCCTATGGATTA
RNAi-4	CCAGAAGAGTTAAGAATCA
lnc-7	
RNAi-1	UAUACUAGGGACUAUGCUG
RNAi-2	UUCCUAGUAAGUAUCUUGG
RNAi-3	AGCGAGCCAGUUUAUCAGGAC
RNAi-4	AUCGUGCUUUGCACAUUGGAC

Supplementary Table 1. The sequences of siRNAs used to knock down each candidate PTEN sp-lncRNA.
